# TSG-SLAM: SLAM Employing Tight Coupling of Instance Segmentation and Geometric Constraints in Complex Dynamic Environments

**DOI:** 10.3390/s23249807

**Published:** 2023-12-13

**Authors:** Yongchao Zhang, Yuanming Li, Pengzhan Chen

**Affiliations:** 1 School of Intelligent Manufacturing, Taizhou University, Taizhou 318000, China; yczhang@tzc.edu.cn; 2 Department of Electrical Engineering, Ganzhou Polytechnic, Ganzhou 341000, China; lym_0111@163.com; 3 School of Electrical and Automation Engineering, East China Jiaotong University, Nanchang 330013, China

**Keywords:** SLAM, complex dynamic environment, fundamental matrix, semantic segmentation, multi-view geometric constraint

## Abstract

Although numerous effective Simultaneous Localization and Mapping (SLAM) systems have been developed, complex dynamic environments continue to present challenges, such as managing moving objects and enabling robots to comprehend environments. This paper focuses on a visual SLAM method specifically designed for complex dynamic environments. Our approach proposes a dynamic feature removal module based on the tight coupling of instance segmentation and multi-view geometric constraints (TSG). This method seamlessly integrates semantic information with geometric constraint data, using the fundamental matrix as a connecting element. In particular, instance segmentation is performed on frames to eliminate all dynamic and potentially dynamic features, retaining only reliable static features for sequential feature matching and acquiring a dependable fundamental matrix. Subsequently, based on this matrix, true dynamic features are identified and removed by capitalizing on multi-view geometry constraints while preserving reliable static features for further tracking and mapping. An instance-level semantic map of the global scenario is constructed to enhance the perception and understanding of complex dynamic environments. The proposed method is assessed on TUM datasets and in real-world scenarios, demonstrating that TSG-SLAM exhibits superior performance in detecting and eliminating dynamic feature points and obtains good localization accuracy in dynamic environments.

## 1. Introduction

SLAM technology is a crucial element for mobile robots to achieve highly intelligent tasks in unknown work environments. Visual SLAM, which relies on visual sensors to perceive surroundings, can acquire images with rich semantic information about environmental targets. Environmental semantic information is of significant importance to intelligent robots as it can assist them in positioning, build environmental semantic maps, and is the basis of human–computer interaction.

In 2007, Davison et al. [1] proposed Mono-SLAM, which achieved the realization of monocular real-time SLAM and initiated research in the field of visual SLAM. Klein et al. [2] proposed PTAM, which creatively divides the entire SLAM system into tracking and mapping threads, successfully applying feature points. Leutenegger et al. [3] proposed the OKVIS visual–inertial odometry framework, while Mur-Artal et al. proposed ORB-SLAM [4], ORB-SLAM2 [5], and ORB-SLAM3 [6] based on feature points.

Most visual SLAM systems are built based on static scenarios, and when there are moving objects in the scenario, the system’s localization and mapping accuracy is greatly affected. In addition, the scene maps constructed by visual SLAM systems are usually based on the geometric information of the scene, such as sparse landmark maps and sparse point cloud maps, which are insufficient to enable mobile robots to understand complex working environments. Thus, it is necessary to process moving objects in complex environments, eliminate their interference in the visual SLAM system, and integrate environmental semantic information to construct semantic maps.

Semantic maps include scene-oriented semantic maps and object-oriented semantic maps. The former integrate semantic information into 3D point clouds to build a scene semantic map, while the latter only retain the semantic information of some objects in the scene semantic map, with most of the semantic information independent of the map in the form of clustering. An object-oriented semantic map is more helpful for a robot to perceive a scene and improve map practicality. McCormac et al. [7] proposed a voxel-based online semantic SLAM system, Hoang et al. [8] proposed the Object-RPE system, and Hosseinzadeh et al. [9] proposed a method to represent objects in the form of quadratic surfaces, while Oberlander et al. [10] proposed a mapping method that combines topological, metric, and semantic information. Hybrid map representation, which combines topological, metric, and semantic information, has been an important direction in the field of mobile robotics research for a long time [11]. Luo et al. [12] used object recognition algorithms to classify scenes, fused the classification results with topological nodes, and assigned semantic information to each topological node. Lin et al. [13] proposed a novel closed-loop approach based on object modeling and semantic graph matching. Object-level features in a scene are modeled using voxels and cuboids, and the scene is further represented as a semantic graph with topological information. Yang et al. [14] proposed a semantic and topological method of automatically representing indoor spaces using floor-plan raster maps to reconstruct indoor spaces with semantic and topological structures. The dynamic visual SLAM method based on the semantic segmentation module proposed by Jin et al. [15] uses semantic labels and depth images to create a 3D point cloud map with semantic information. In short, the fusion of topological and semantic information generally only fuses semantic information with topological nodes, ignoring many environmental details. Although it can help a mobile robot to move to a certain scene quickly, it cannot allow the robot to intelligently interact with the physical objects in the scene.

The rest of this paper is organized as follows: Section 2 provides an overview of related works regarding SLAM methods in dynamic environments. Section 3 describes the proposed system framework. Section 4 presents a dynamic feature removal method in detail. Section 5 presents the semantic map construction module. Section 6 presents the experimental results and performance analysis. Finally, Section 7 concludes the paper and discusses future research directions.

## 2. Related Work

Semantic SLAM systems face challenges in accurately localizing and mapping complex environments with a large number of dynamic objects. To address this issue, four methods [16] have been proposed to eliminate dynamic features: multi-sensor information compensation, an enhanced RANSAC algorithm, foreground/background model construction, and semantic information integration.

Using information obtained from an IMU, a wheel odometer, and other sensors as prior motion knowledge for a camera can assist a system in segmenting dynamic targets. The SLAM system designed by Yao et al. [17] includes tracking threads, feature extraction threads, and local mapping threads. One of the tasks of the tracking thread is to utilize the transformation matrix obtained from an IMU and combine it with the reprojection error to determine the dynamic nature of feature points. To avoid long-term drift in wheel odometry calculations, Yang et al. [18] only used data between two adjacent frames to estimate the initial pose over a short period of time. In order to speed up the detection of dynamic regions, two optimization measures were adopted. First, the dynamic nature of the image regions obtained via clustering was determined instead of individual pixels. Second, when judging the clustered regions, only a subset of feature points was selected instead of all feature points in the region. In addition, the object detection framework YOLOv3 [19] was integrated into the system. The data fusion and joint calibration resulting from the use of multiple sensors pose challenges to the system’s stability and accuracy, while errors and noise are also issues that cannot be neglected.

For SLAM in dynamic environments, it is common to use the RANSAC algorithm to obtain a rough transformation matrix result to determine the dynamic status of landmarks in the environment. Sun et al. [20] used tthe RANSAC algorithm to compute a perspective transformation. The calculated transformation matrix was applied to each pixel in the previous frame image, resulting in a difference image. The moving object can be roughly identified using the non-zero pixels in the differencing results. This process was used to obtain segmentation results. Sun et al. [21] replaced the standard RANSAC algorithm with a least-median-of-squares algorithm (LMedS) for their calculations. The Prior-based Adaptive Random Sample Consensus (PARSAC) algorithm proposed by Tan et al. [22] leveraged prior knowledge about the background, leading to a reduced proportion of inliers in the estimation of the camera’s motion model. The major drawback of using the RANSAC algorithm to eliminate dynamic objects is that as the number of dynamic objects increases or they become closer to the camera, the static background area becomes too small, leading to ineffective dynamic object removal.

Building a foreground model in advance is equivalent to directly segmenting out moving objects. Then, the areas outside the foreground are used for the localization and mapping of mobile robots. Wang et al. [23] first calculated optical flow trajectories between consecutive image frames. They performed a clustering analysis on these trajectories, merging regions with similar motion tendencies. Assuming that static regions dominate the majority of the image, the largest area with merged regions could be used to compute the corresponding fundamental matrix. This process further refined the dynamic regions, forming the image foreground. In subsequent camera localization and dense mapping using the Dense Visual Odometry (DVO) SLAM system [24], the foreground parts of the images were discarded. The challenge in constructing foreground models lies in the identification and removal of non-rigid bodies, such as pedestrians and animals.

Adding semantic information to the SLAM system in dynamic environments allows for the preliminary assessment and segmentation of objects with high motion probability based on prior knowledge. By removing these high-motion-probability target regions from the images, estimating the camera’s motion and pose becomes much more reliable compared to directly using the RANSAC algorithm to remove outliers. Yu et al. [25] introduced the DS-SLAM system, which excluded the person area in the image and eliminated dynamic matching points using motion consistency. Bescos et al. proposed DynaSLAM [26] and DynaSLAM II [27]. DynaSLAM combined multi-view geometry and target masks to remove predefined moving objects and proposed a background restoration method to fix occluded backgrounds. Dyna-SLAM II simultaneously estimated camera poses, sparse static 3D maps, and the trajectories of multiple moving objects using a new bundle adjustment method. You et al. [28] proposed a multimodal semantic SLAM system (MISDSLAM) which can reconstruct the static background with semantic information. Liu et al. [29] applied an algorithm to obtain as the latest semantic information possible, thereby making it possible to use segmentation methods with different speeds in a uniform way. Zhao et al. [30] proposed KSF-SLAM, which added an efficient semantic tracking module to remove dynamic objects in dynamic environments. Gonzalez et al. [31] introduced TwistSLAM, which created point clusters based on semantic categories and modeled constraints between clusters to remove dynamic features and improve motion estimation quality. Kuang et al. [32] obtained potential motion areas through semantic segmentation, combined dynamic point features to determine dynamic areas, and removed point and line features in dynamic areas to enhance localization accuracy and stability. Runz et al. [33] presented the mask fusion system, which used geometric segmentation to produce precise object boundaries to overcome the limitations of imperfect boundaries provided by semantic segmentation. Xu et al. [34] proposed the MID-Fusion system, which provided the geometric, semantic, and motion attributes of objects in an environment. Li et al. [35] presented the DP-SLAM system, which tracked dynamic matching points in a Bayesian probability estimation framework to overcome geometric constraints and semantic segmentation bias. Wu et al. [36] proposed YOLO-SLAM, which combined object detection and geometric constraint methods to reduce the influence of dynamic objects. Li et al. [37] utilized semantic information and global dense optical flow as constraints to generate dynamic-static masks and eliminate dynamic objects. Xing et al. [38] presented DE-SLAM, which utilized a dynamic detection and tracking module of semantic and metric information to improve localization accuracy by eliminating features on dynamic objects.

In the aforementioned literature, most dynamic visual SLAM schemes adopted existing mature object detection and semantic segmentation network frameworks to perform the initial division of dynamic regions. The network architectures included SegNet [39], Mask R-CNN [40], and the YOLO series. Similar to Mask R-CNN, SOLOv2 [41] is a simple, fast, and accurate instance segmentation framework. It surpasses most current advanced open-source instance segmentation methods in terms of segmentation speed and accuracy, and it also performs well in segmenting moving targets. Hence, we chose SOLOv2 to complete the instance segmentation task. Based on instance-level semantic information, we can acquire the prior motion information of objects. If the label corresponding to the object feature is person, it is considered dynamic, and if the label is desk, it is considered static, both with high confidence. However, when the label is chair, it is usually static, but there is a significant likelihood it might move due to the influence of other moving objects (such as human activity). Therefore, it is challenging to definitively categorize chairs as either static or dynamic; these are potentially dynamic objects.

In conventional semantic segmentation-based dynamic SLAM systems, dynamic features are removed, static features are preserved, and potentially dynamic features are generally treated either as all static or as all dynamic. Treating all potentially dynamic features as dynamic and removing them can reduce the accuracy of feature matching. Conversely, treating all potentially dynamic features as static can lead to many incorrect correspondences in feature matching. Both situations negatively affect the system’s localization accuracy and mapping precision. In essence, while the SOLOv2 algorithm can segment potential dynamic targets and provide semantic labels, it cannot accurately determine their actual motion state. In addition, the unavoidable fuzziness in SOLOv2’s segmentation results near object edges can lead to a small number of feature points being misjudged at the edges where dynamic and static objects meet. Therefore, we cannot rely solely on SOLOv2’s semantic information and need to combine it with other methods to jointly determine the motion state of target features.

Instance segmentation methods are used in conjunction with other methods, such as using a bundle adjustment with multi-view geometric constraints or optical flow fields. When combined, a voting mechanism is typically employed to process dynamic objects. Generally, there are two types of voting mechanism: the first is that if both judgment results are dynamic, the final result is dynamic, and the second is that if any one result is dynamic, the final result is dynamic. We consider both combination methods loosely coupled approaches, merely combining the results of the two methods through a simple mechanism. In fact, this loosely coupled approach is unreliable and can lead to misjudgment.

This paper proposes a dynamic feature removal method that tightly couples instance segmentation and multi-view geometric constraints to detect and remove dynamic feature points and integrates instance semantic information into environment map construction to generate global environment instance-level semantic point cloud maps. The main contributions of this paper are as follows. First, a system framework for the SLAM of mobile robots in complex environments is constructed based on ORB-SLAM3. Second, a dynamic feature removal method is designed which uses a tightly coupled method to closely combine the instance segmentation SOLOv2 algorithm with multi-view geometric constraints to accurately detect and remove dynamic feature points. Third, a semantic map construction module is designed, which extracts a 3D semantic point cloud using the semantic information of the target obtained via the instance segmentation algorithm, generates the corresponding target semantic tag, and builds an instance-level target semantic tag library to construct an environmental 3D semantic map.

## 3. System Overview

**Figure 1:**
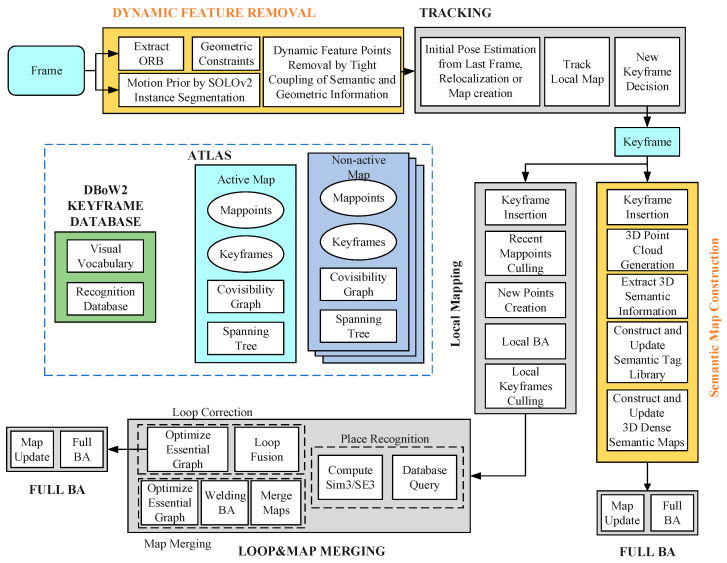
Overall SLAM system framework.

To eliminate the impact of dynamic targets on the SLAM system and create a map containing environmental semantic information, this paper presents a mobile robot simultaneous localization and semantic mapping system based on the ORB-SLAM3 system. The system’s overall framework is illustrated in Figure 1, and it can handle dynamic targets with excellent anti-interference ability, extract the instance-level semantic information of various objects, and support intelligent robots to perform tasks in complex indoor environments.

The TSG-SLAM system introduces two additional parallel threads to the classic three threads of ORB-SLAM3: the dynamic feature removal thread and the semantic map construction thread. The dynamic feature removal thread is responsible for eliminating the dynamic features of objects, ensuring the system’s localization and mapping accuracy. The semantic map construction thread constructs a 3D dense semantic map with instance-level semantic information, which enables intelligent robots to navigate and interact intelligently in complex environments.

## 4. Dynamic Feature Removal Method

**Figure 2:**
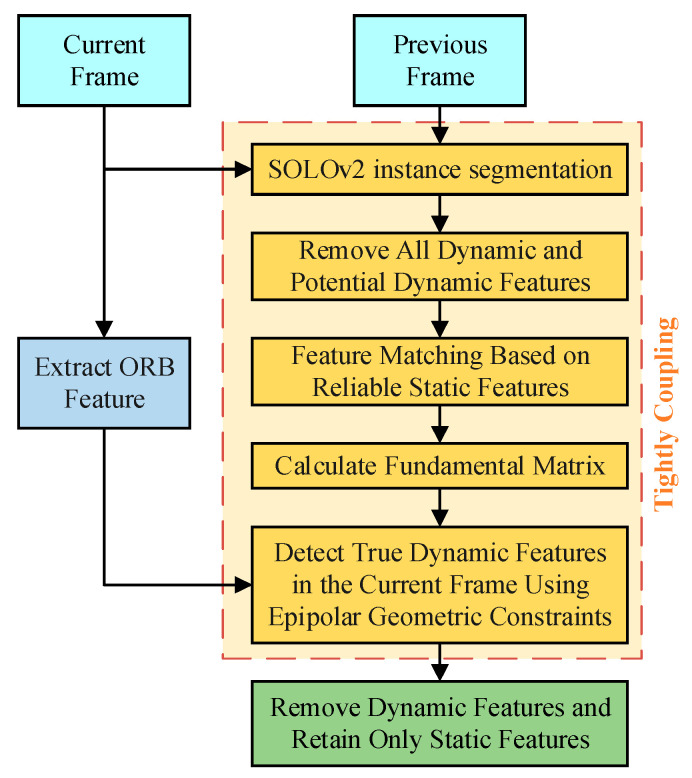
Framework of dynamic feature removal algorithm.

A dynamic feature detection and removal method is proposed in this paper. The method tightly integrates semantic information and multi-view geometric constraint information, as shown in the algorithm framework in Figure 2.

Firstly, ORB features are extracted from the current frame image, and instance segmentation results are obtained using SOLOv2 on both the current and previous frames. This allows for the removal of features belonging to dynamic targets and potential dynamic targets. Subsequently, feature matching is performed based on the remaining static targets, and the fundamental matrix is calculated. Finally, dynamic feature points in the current frame are precisely detected and removed with multi-view geometric constraints, leaving only the static feature points.

This method ensures accurate localization and mapping in complex environments by removing the impact of dynamic targets and contributes to constructing a 3D dense semantic map with instance-level semantic information for intelligent tasks such as navigation and interaction in complex indoor environments.

### 4.1. SOLOv2 Instance Segmentation

**Figure 3:**
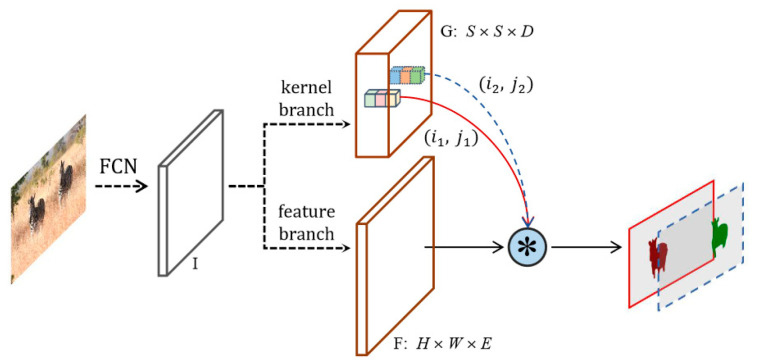
Framework of SOLOv2 network.

The SOLOv2 network architecture is shown in Figure 3, and it mainly comprises Fully Convolutional Network (FCN) feature extraction, a kernel branch, and a feature branch. The convolution kernel matrix is denoted as G, while the mask feature matrix is represented by F. SOLOv2 divides the image into S × S grids, treating each grid as a potential target instance. After the original image is passed through the FCN, the feature map is obtained, which then enters both the kernel branch and the feature branch. The kernel branch predicts the dynamic kernel to obtain different kernels for different inputs, while the feature branch predicts the features for each point on the feature map. Finally, the outputs of the kernel branch and feature branch are convolved to obtain the mask of the target in the image.

The COCO dataset [42] is used for pre-training to obtain network parameters, which include most moving objects that may appear in real-life scenarios, making it very suitable for the application scenario of this article.

### 4.2. Dynamic Feature Detection

The multi-view geometry constraints utilizing epipolar geometry characteristics can be used to detect the motion state of target feature points in the environment. The features that satisfy the epipolar constraints are static features, while the features that do not satisfy the epipolar constraints are dynamic features. These constraints can be used to identify the position and motion of feature points in a given environment.

**Figure 4:**
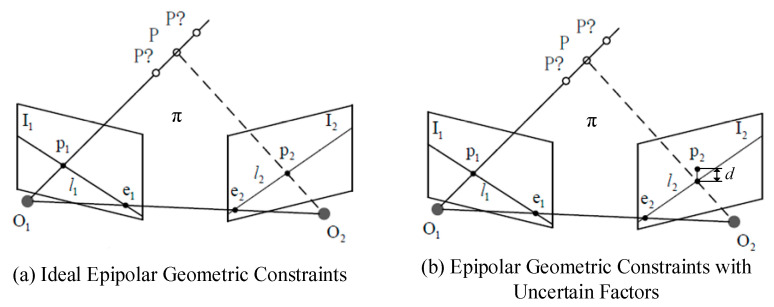
Multi-view epipolar geometry constraints.

Figure 4a shows the relationship between static object points and their corresponding feature points in two frames. *P* is a static target point which is imaged in two consecutive image frames corresponding to feature points *p*_1_ and *p*_2_ in frame *I*_1_ and frame *I*_2_, respectively. *P*? represents the possible position of point *P* in the presence of uncertain factors. *O*_1_ and *O*_2_ are the camera centers corresponding to frame *I*_1_ and frame *I*_2_, respectively. Polar plane π intersects image planes *I*_1_ and *I*_2_ at polar lines *l*_1_ and *l*_2_, respectively, and baseline *O*_1_*O*_2_ intersects image planes *I*_1_ and *I*_2_ at poles *e*_1_ and *e*_2_, respectively. *P* lies on rays $\vec{O_{1}p_{1}}$ and $\vec{O_{2}p_{2}}$, and *p*_2_ lies on epipolar line *l*_2_. The multi-view geometric constraint describes the corresponding epipolar mapping from the points on frame *I*_1_ and frame *I*_2_, and the mapping relationship can be described by the fundamental matrix *F_m_*.

(1) $$p_{2}^{T}F_{m}p_{1}=0$$

Given *p*_1_ in frame *I*_1_ and the fundamental matrix *F_m_*, Equation (1) provides the constraints that *p*_2_ must satisfy when *P* is a static target point. Therefore, we can use this constraint to judge whether the target point corresponding to the ORB feature point is dynamic. Due to the uncertainty in the process of extracting features and estimating *F_m_*, there is a high probability that the two image points in the static map do not strictly satisfy Equation (1), that is, *p*_2_ in Figure 4b should be located on *l*_2_. If the distance *d* between *p*_2_ and *l*_2_ is smaller than a predetermined threshold, the motion state of the target point corresponding to the image point is regarded as static; otherwise, it is regarded as dynamic.

Use the classic eight-point algorithm to estimate the fundamental matrix *F_m_.* Let $F_{m}=\left(\begin{array}{ccc} f_{1} & f_{2} & f_{3}\\ f_{4} & f_{5} & f_{6}\\ f_{7} & f_{8} & f_{9} \end{array}\right)$, $p_{1}={\left[u_{1},v_{1},1\right]}^{T}$,$p_{2}={\left[u_{2},v_{2},1\right]}^{T}$, where $\left(u_{1},v_{1}\right)$ and $\left(u_{2},v_{2}\right)$ are the pixel coordinates of *p*_1_ and *p*_2_, respectively. According to Equation (1), we can obtain

(2) $$\left(u_{2},v_{2},1\right)\left(\begin{array}{ccc} f_{1} & f_{2} & f_{3}\\ f_{4} & f_{5} & f_{6}\\ f_{7} & f_{8} & f_{9} \end{array}\right)\left(\begin{array}{c} u_{1}\\ v_{1}\\ 1 \end{array}\right)=0$$

Let *f_m_* denote the vector containing all elements of *F_m_*.

(3) $$f_{m}={\left[f_{1},f_{2},f_{3},f_{4},f_{5},f_{6},f_{7},f_{8},f_{9}\right]}^{T}$$

Equation (2) can be written as a linear equation about *f_m_*.

(4) $$\left[u_{2}u_{1},u_{2}v_{1},u_{2},v_{2}u_{1},v_{2}v_{1},v_{2},u_{1},v_{1},1\right]\cdot f_{m}=0$$

When there are eight pairs of corresponding image points between two consecutive frames, we can solve Equation (4) to calculate *F_m_*. Once *F_m_* is obtained, we can use Equation (2) to determine the state of the target feature.

While using multi-view epipolar geometry constraints to detect dynamic features is a useful approach, it presents a fundamental contradiction. In order to calculate the fundamental matrix *F_m_* required to detect dynamic feature points, correspondences of static feature points are needed as the target features in the keyframes used for feature matching must be static. This means that dynamic feature points must be removed before *F_m_* can be calculated. In the general feature-matching process, the Random Sample Consensus (RANSAC) algorithm is often used to filter out dynamic feature points and reduce the impact of incorrect correspondences. However, RANSAC is limited in its ability to remove a large number of dynamic feature points, which can negatively affect its overall performance.

### 4.3. Dynamic Feature Removal

We propose a tight-coupling approach that utilizes the fundamental matrix *F_m_* as a bridge between semantic information and geometric constraint information. Firstly, we employ SOLOv2 to perform instance segmentation on frames of the scenario to obtain motion priors, which identify all moving and potential moving targets. We then use the instance segmentation results as a mask to remove the correspondences of dynamic and potential dynamic features, retaining only reliable static feature correspondences. Based on these static feature correspondences, we perform feature matching and compute the reliable fundamental matrix. Finally, we use multi-view geometry constraints to detect and remove true dynamic features, retaining only static feature points for subsequent tracking and mapping. When judging the motion state of a feature point, we use a threshold value of *d*. If *d* exceeds one pixel size, the feature point is judged as dynamic and removed, and if *d* is smaller than a pixel size, it is judged as static and retained.

## 5. Instance-Level Semantic Map Construction

**Figure 5:**
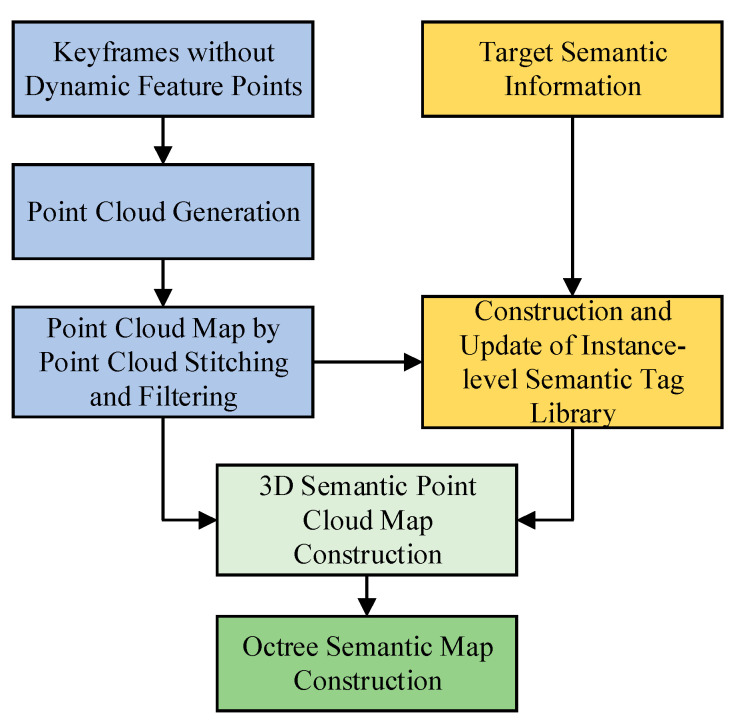
Framework of scenario semantic map construction algorithm.

Figure 5 illustrates the framework of our scenario semantic map construction algorithm. The algorithm constructs the semantic map using keyframe images with dynamic feature points removed. Firstly, we generate a single-frame point cloud containing only static feature points from the keyframe images. Then, we stitch and filter the generated single-frame point clouds to obtain the scene point cloud map. Next, we use the semantic information and masks provided by SOLOv2 to extract the 3D semantic tags of the targets from the point clouds, establishing and updating an instance-level semantic tag library. Finally, we integrate the semantic information of the targets into the point cloud map to generate a 3D semantic point cloud map. To accommodate larger scenes and conserve storage space, we construct an octree semantic map.

An RGB-D camera captures both color and depth information for each pixel in the scene. By modeling the camera and using its intrinsic and extrinsic parameters, we can map the 2D pixels in the image to their corresponding 3D points in space, creating a point cloud. For a given frame, let (*x*, *y*) be the 2D coordinates of a pixel *p*, (*X*, *Y*, *Z*) be the 3D coordinates of the corresponding spatial point *P*, and *s* be the depth value of *p*. The transformation relationship between *p* and *P* can be expressed as follows:

(5) $$\left[\begin{array}{c} X\\ Y\\ Z \end{array}\right]={\left[\begin{array}{ccc} f_{x} & 0 & c_{x}\\ 0 & f_{y} & c_{y}\\ 0 & 0 & 1 \end{array}\right]}^{-1}\left[\begin{array}{c} x\\ y\\ s \end{array}\right]$$

The coordinates of *P* are

(6) $$\left\{\begin{array}{c} X=s\left(x-c_{x}\right)/f_{x}\\ Y=s\left(y-c_{y}\right)/f_{y}\\ Z=s \end{array}\right.,$$

where *f_x_* and *f_y_* are the focal length of the RGB-D camera, and *c_x_* and *c_y_* are the offsets of the image origin relative to the imaging point of the camera’s optical center. By applying this transformation to the pixels in the key image frames, we can obtain the corresponding point cloud.

To match and stitch point clouds, we utilize the PCL (Point Cloud Library) [43] and follow a three-step process. First, we find the point cloud that corresponds to a certain frame and match them. Second, we calculate the transformation matrix between the two point clouds. Finally, we transform the matched point clouds into the same coordinate system and stitch them together, resulting in a complete point cloud map of the scene.

The mathematical expression for point cloud stitching can be described as follows:

(7) $$m=\sum_{i=0}^{n}T_{i}C_{i},$$

where *m* is the local point cloud map obtained by generating and stitching the first *n* image frames. *C_i_* represents the point cloud obtained from the *i*-th keyframe, and *T_i_* represents the position and orientation of the camera corresponding to the *i*-th keyframe.

To remove outlier noise points from the point cloud map, a statistical filter is employed to filter the point cloud map to remove these outlier noise points. To address the issue of overlapping points obtained from different viewing angles while preserving the shape characteristics of the point cloud map, a voxel filter is used to remove these overlapping points.

Although we have generated a global 3D point cloud map of the scene through single-frame point cloud generation, point cloud stitching, and filtering, this point cloud map is simply geometry-based and does not incorporate the semantic information of targets. As a result, it cannot provide a deeper understanding of the scene for mobile robots. Therefore, we designed an algorithm for constructing and updating an instance-level Semantic Tag Library (STL), presented in Algorithm 1. Firstly, we extract and optimize the 3D point cloud corresponding to each target to generate the corresponding 3D semantic tag. Then, we match and fuse the semantic tags corresponding to the 3D point clouds extracted from the same target in different perspectives. Finally, we construct and update the global static target semantic tag library of the scene.
**Algorithm 1** Algorithm of instance-level STL.**Input**: semantic information and 3D point cloud of targets**Output**: instance-level STL1 **for** each 3D point cloud **do**2  **if** an unprocessed point cloud exists **then**3   extract & optimize point cloud for semantic tag4     **if** semantic tag exists in tag library **then**5      calculate spatial consistency of point cloud6      **if**
*d*_min_ < *d*_w_ **then**7               fuse & update STL8               go to step 189      
**else**
10             insert the tag into STL11             go to step 1812          **end**
13         
**else**
14            insert the tag into STL15            go to step 1816         
**end**
17         
**else**
18            save STL19         
**end**
20  
**end for**


Since the SOLOv2 instance segmentation algorithm can accurately segment the target area, the resulting target mask area is highly precise and contains only pixels corresponding to the target. This makes it possible to map the target semantic information obtained from the segmentation directly to the 3D point cloud, resulting in a 3D point cloud corresponding to each target. The detailed process involves locating the region of each segmented target instance using the 2D mask, recording the index of the corresponding point cloud for each pixel in the mask area that matches the semantic mask category, calculating the average depth of the point cloud in the target mask area, removing outlier points, and performing statistical and voxel filtering on the point cloud index corresponding to each target. Instance-level semantic tags are then generated based on the semantic information and corresponding 3D point clouds.

To update the target semantic tag library, the target semantic tags generated from the segmentation are compared with existing tags in the library. If a tag with the same category does not exist, it is added to the library. If it does exist, a spatial consistency calculation is performed on the 3D point cloud, and if the minimum Euclidean distance *d*_min_ between the centers of the point clouds is less than the average width *d*_w_ of the two candidate boxes, they are considered the same target, and the target semantic tag library is fused and updated. Otherwise, the target semantic tag is inserted into the library. The library updating process involves merging similar targets’ point clouds and recalculating their center, maximum, and minimum point coordinates.

After constructing and updating the semantic tag library, the 3D point cloud map contains the instance-level semantic information of each target in the scenario. However, it also contains invalid information, such as textures on the ground and shadows in shadowed areas, which could overload the computing resources. Therefore, to achieve the localization and mapping of larger-scale scenes, a visual 3D octree semantic map is established by performing point cloud semantic extraction on each keyframe and matching and fusing target point clouds generated from different observations at different positions.

Suppose a certain node in the map is denoted as *n*, its observed value is *z*, and the probability log value of the node from the beginning to the time *t* is $L\left(n\left|z_{1:t}\right.\right)$; then, the probability log value at the time *t* + 1 is

(8) $$L(n\left|z_{1:t+1}\right.)=L(n\left|z_{1:t-1}\right.)+L(n\left|z_{t}\right.),$$

written in probabilistic form as

(9) $$P(n\left|z_{1:t}\right.)={\left[1+\frac{1-P(n\left|z_{t}\right.)}{P(n\left|z_{t}\right.)}\cdot \frac{1-P(n\left|z_{1:t-1}\right.)}{P(n\left|z_{1:t-1}\right.)}\cdot \frac{P(n)}{1-P(n)}\right]}^{-1}$$

With the help of log probabilities, we can effectively combine and enhance the entire octree map using RGB-D data. When the depth information of each pixel is converted into point cloud data, the 3D point cloud representing the target will be contained within the limits of the corresponding octree sub-node. By increasing the occupancy probability of that node, we can obtain the occupancy information of the node. Furthermore, by assigning target semantic RGB color values to each node of the octree, we can create a highly visual octree semantic map.

## 6. Experiment and Discussion

### 6.1. Test of Dynamic Feature Removal Method

**Figure 6:**
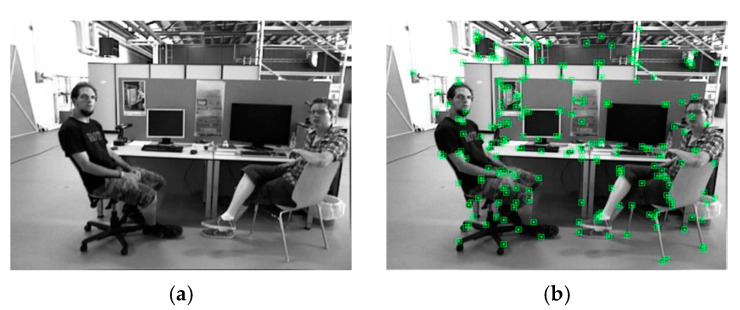

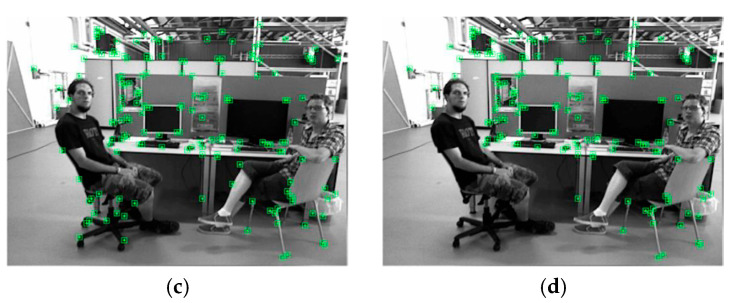
Comparison of dynamic feature removal method. (**a**) Original grayscale image. (**b**) ORB feature extraction. (**c**) Dynamic feature removal based on instance segmentation. (**d**) Dynamic feature removal based on tightly coupled method.

To analyze the effectiveness of the dynamic feature removal method, we selected the fr3/walking_xyz dataset from the TUM dataset [44] for testing. This dataset scenario is similar to our daily work environment, as shown in Figure 6a, with static objects such as tables and monitors, dynamic targets such as people, and potential dynamic targets such as chairs. Two monitors are static, two people are dynamic, and the chair on the left moves due to the movement of the person and is dynamic, while the chair on the right has not been moved and is static. Figure 6b presents the ORB feature extraction results, with feature points marked with green dots distributed throughout the scenario. Many features are extracted from objects with distinctive features such as people, chairs, and monitors, which contain numerous dynamic features, such as people. In Figure 6c, we show the results of removing dynamic features only with the SOLOv2 instance segmentation method. Although most of the ORB dynamic features on the two people are removed, a few feature points remain at the contact edge between the people and the chair, limited by the accuracy of the instance segmentation algorithm. It is difficult to perfectly segment features at a contact edge, and the SOLOv2 algorithm used in this paper, despite having high segmentation accuracy for object edges, still has some unavoidable errors. Additionally, the feature points on the two chairs are not removed since the real state of the potential dynamic target cannot be accurately distinguished only based on instance segmentation. Therefore, the two chairs are simply judged as static targets. Figure 6d illustrates the results of our tightly coupled method for removing dynamic features. Compared to Figure 6c, almost all the features of the two moving people are removed, indicating that our method is more effective at reducing the segmentation error of the instance segmentation algorithm. The features of the chair on the right are judged as static features and preserved, while the features of the chair on the left are judged to be dynamic and removed, indicating that our method accurately removes the features of all dynamic objects in the scenario. This is consistent with the motion state of each target in the test dataset. Table 1 lists the number of different types of feature points obtained by different methods.

Our findings confirm the effectiveness of our method in accurately eliminating dynamic features and minimizing segmentation errors in instance segmentation algorithms.

### 6.2. Test of Semantic Map Construction Algorithm

**Figure 7:**
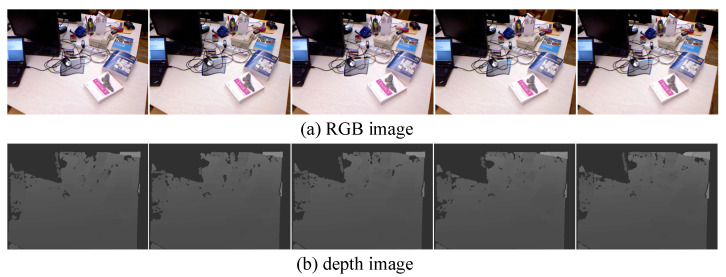
Selected image frame sequence diagram.

We utilized the partial sequence located in the fr1/room of the TUM dataset to conduct our local semantic mapping evaluation. This dataset provides us with RGB images, depth images, and the precise position and orientation of the camera. For our mapping test, we selected five consecutive frames of images. Figure 7a shows the RGB images of the selected frames, while Figure 7b displays the corresponding depth images.

**Figure 8:**
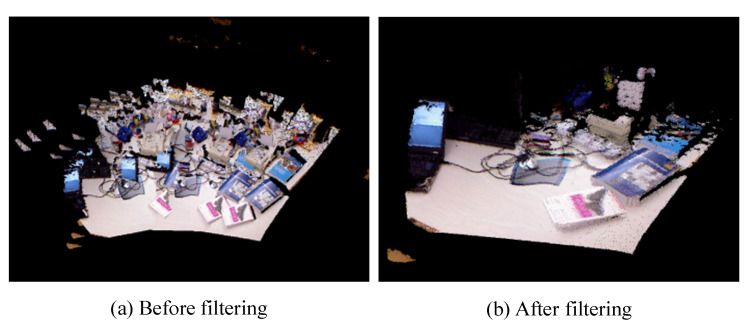
Comparison of point cloud map before and after filtering.

To create our point cloud map, we stitched and filtered all of the single-frame point clouds generated from extraction. The point cloud map before and after filtering is depicted in Figure 8, where Figure 8a,b illustrate the point cloud map before and after filtering, respectively. Prior to filtering, the point cloud map exhibited a significant amount of overlap between point clouds and contained numerous outlier noise points. However, after filtering, the quality of the point cloud map was significantly enhanced. Our experimental statistical analysis revealed that the numbers of point clouds generated before and after filtering were 1,118,657 and 634,787, respectively, representing a reduction of almost half of the total number of point clouds. This highlights how filtering can effectively improve mapping outcomes and greatly conserve computing resources.

**Figure 9:**
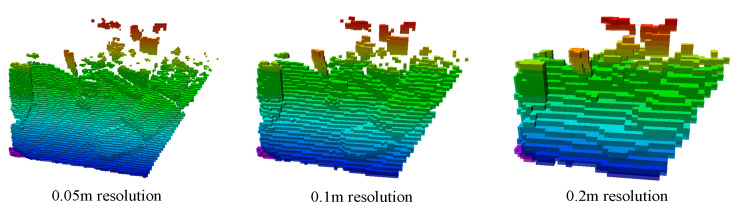
Octree map before integrating target semantic color information.

**Figure 10:**
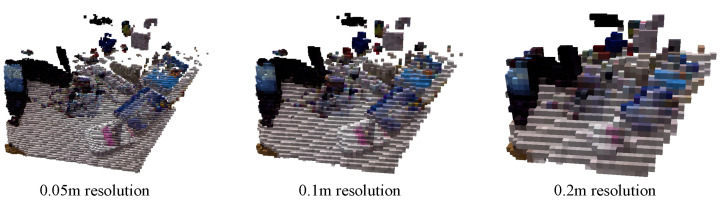
Octree map after integrating target semantic color information.

Figure 9 and Figure 10 display the reconstruction results of the octree maps before and after integrating target semantic color information. In Figure 9, the octree map is annotated with a gradient color scheme without a specific pattern. In Figure 10, the octree map with the added target semantic color information contains visualized semantic information of the scenario’s targets in Figure 8b, which significantly enhances the scenario reconstruction and produces a visually compelling result. Additionally, Figure 9 and Figure 10 demonstrate the impact of the octree map at varying resolutions. In our testing, we used a default depth of 16 layers, with an edge length of each small square measuring 0.05 m. As the depth decreases by one layer, the leaf nodes of the octree move up one layer, and the edge length of each small square doubles.

Moreover, the file sizes of the point cloud map and the octree map are 10.2 megabytes and 217.8 kilobytes, respectively. This represents a significant reduction in storage space of nearly fifty times, highlighting the benefits of using an octree map for reconstructing larger scenarios.

### 6.3. Experimental Platform and Evaluation Index

To test the feasibility and effectiveness of our SLAM system in complex environments with dynamic objects, we conducted experiments in static, low-dynamic, and high-dynamic public datasets, as well as in real laboratory scenarios. In order to simplify subsequent discussions, we gave our improved SLAM system a name: TSG-SLAM.

**Figure 11:**
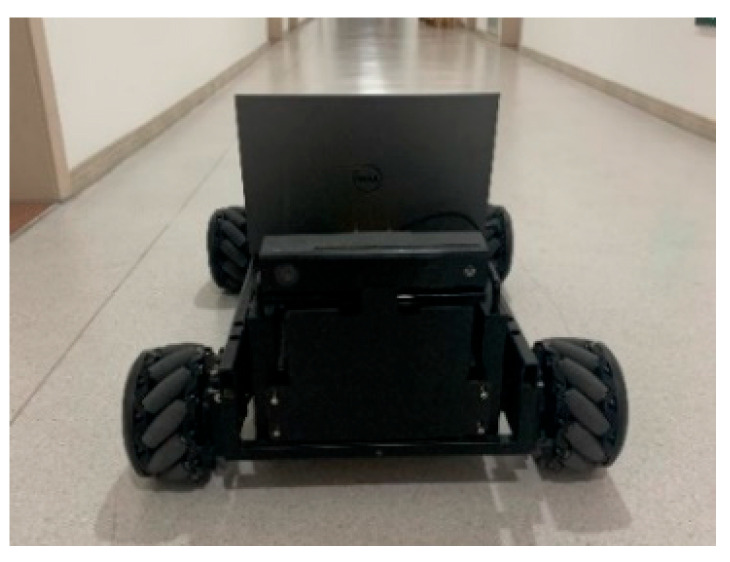
Experimental platform.

An experimental platform for mobile robots was developed to meet the demands of complex environments. It includes a Mecanum wheel mobile robot, a Kinect V2 depth camera (Microsoft Inc., Redmond, WA, USA), a computer, and a vehicle-mounted power supply, among other components. This platform is depicted in Figure 11.

The software system is built on Ubuntu 16.04 and utilizes the ROS system for managing the entire system. The program is primarily written in the C++ language and utilizes various open-source libraries, including OpenCV for processing keyframe images, Eigen for matrix operations, Keras for instance segmentation, Ceres for solving least squares problems during optimization, g2o for graph optimization, PCL for generating point clouds, and octomap [45] for constructing octree maps.

To assess the localization accuracy of a SLAM system, the absolute trajectory error (ATE) and the relative pose error (RPE) are used as evaluation metrics to evaluate the motion trajectory estimation. ATE is employed to assess the overall accuracy of the SLAM system. The formula for calculating ATE is as follows:

(10) $$\mathrm{ATE}=\sqrt{\frac{1}{N}\sum_{i=1}^{N}∥trans(T_{g,i}^{-1}T_{e,i}){∥}_{2}^{2}},$$

where *N* is the number of frames, and $T_{g,i}$ and $T_{e,i}$ are the true position value and evaluated position value of the *i*-th frame.

The RPE metric is utilized to assess the local accuracy of the trajectory estimation and the position estimation drift of the SLAM system within a certain fixed time. Within a fixed time interval *t*, RPE can be obtained as follows:

(11) $$\mathrm{RPE}=\sqrt{\frac{1}{N-\Delta t}\sum_{i=1}^{N-\Delta t}∥trans({\left(T_{g,i}^{-1}T_{g,i+\Delta t}\right)}^{-1}(T_{e,i}^{-1}T_{e,i+\Delta t})){∥}_{2}^{2}}$$

where Δ*t* represents the number of frames within *t*.

### 6.4. Public Dataset Experiments

To test the system, static, low-dynamic, and high-dynamic scenarios were chosen from the TUM dataset. The selected static scenarios were fr1/desk and fr1/room. The fr1/desk sequence had a smaller camera movement range, capturing mainly indoor local scenarios focused on the table and items on it. The fr1/room sequence, on the other hand, had a larger camera movement range and included most indoor spatial scenarios. To better analyze the impact of dynamic objects on localization and mapping, dynamic datasets were selected with dynamic targets as the primary subject. The low-dynamic scenarios were fr3_sitting_static and fr3_sitting_xyz, which depicted two people sitting in chairs and talking, accompanied by small movements such as waving and turning their heads. The fr3_sitting_static camera had a small range of motion, while the fr3_sitting_xyz camera had a large range of motion around the dynamic subject in the x-y-z direction. The high-dynamic scenarios were fr3_walking_static and fr3_walking_xyz, depicting two people with fast and large-scale movements. The camera movements in fr3_walking_static and fr3_walking_xyz were similar to those in fr3_sitting_static and fr3_sitting_xyz, respectively.

To compare TSG-SLAM’s performance with ORB-SLAM3, we conducted an analysis of ATE and RPE data, including the mean, median, root mean square error (RMSE), and standard deviation (STD). The RMSE measures the precision of the observed values, which reflects the accuracy of the system, while the STD measures the dispersion of the observed values, which reflects the robustness of the system. Moreover, we also calculated the improvement rate of TSG-SLAM’s localization performance relative to ORB-SLAM3 by using the formula below:

(12) $$\eta =\frac{\delta_{1}-\delta_{T}}{\delta_{1}}\times 100\%,$$

where η is the improvement rate (IR) and $\delta_{T}$ and $\delta_{1}$ are the error of TSG-SLAM and ORB-SLAM3, respectively.

Table 2 illustrates a comparison of ATE and RPE in static scenarios. As can be observed from the table, both systems have small errors in all aspects for the local scenario fr1/desk. In the global scenario fr1/room, ATE increases significantly but still within an acceptable range, and the errors of the two systems are very close, with some errors being lower than ORB-SLAM3. ORB-SLAM3 is presently one of the most mature visual SLAM algorithms known for its high localization accuracy in static scenarios. TSG-SLAM introduces a dynamic feature removal module based on ORB-SLAM3, and its effect is not significant in static scenarios. Thus, the localization accuracy of both systems in static scenarios is quite similar. 

**Figure 12:**
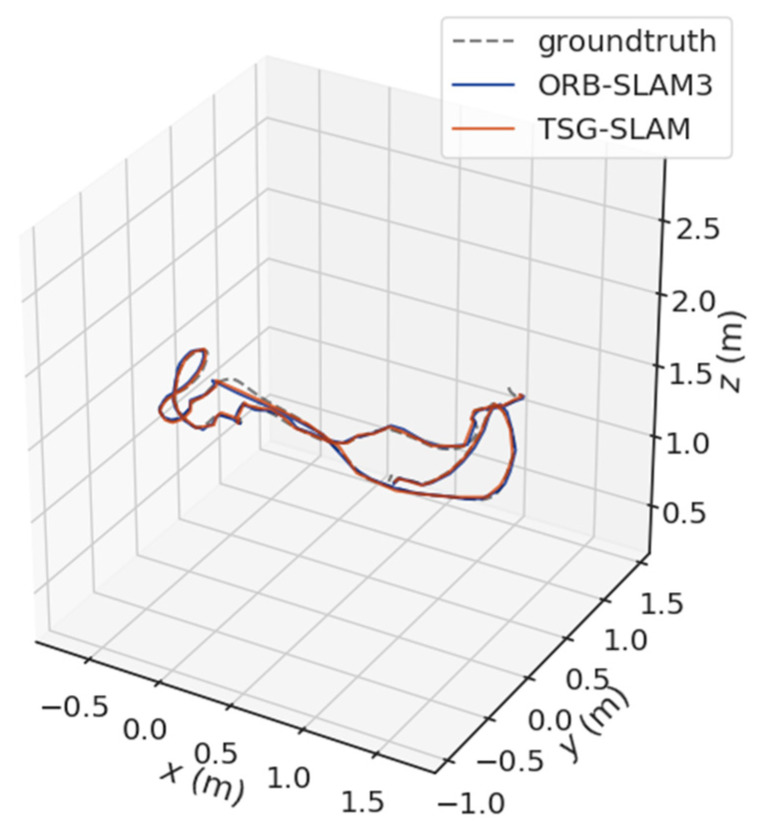
Estimated trajectory vs. true trajectory for fr1/desk.

**Figure 13:**
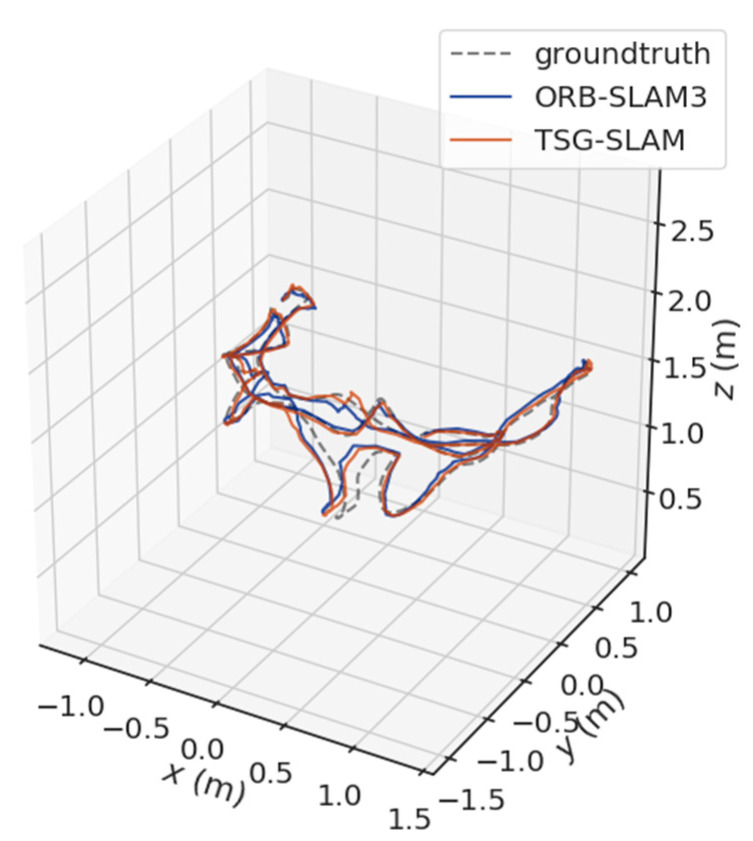
Estimated trajectory vs. true trajectory for fr1/room.

Figure 12 and Figure 13 show a comparison of estimated trajectories and true trajectories for the fr1/desk and fr1/room sequences, respectively. The estimated trajectory closely follows the true trajectory. Therefore, in static scenarios, TSG-SLAM does not have a significant advantage in localization performance, and both systems exhibit high localization accuracy.

**Figure 14:**
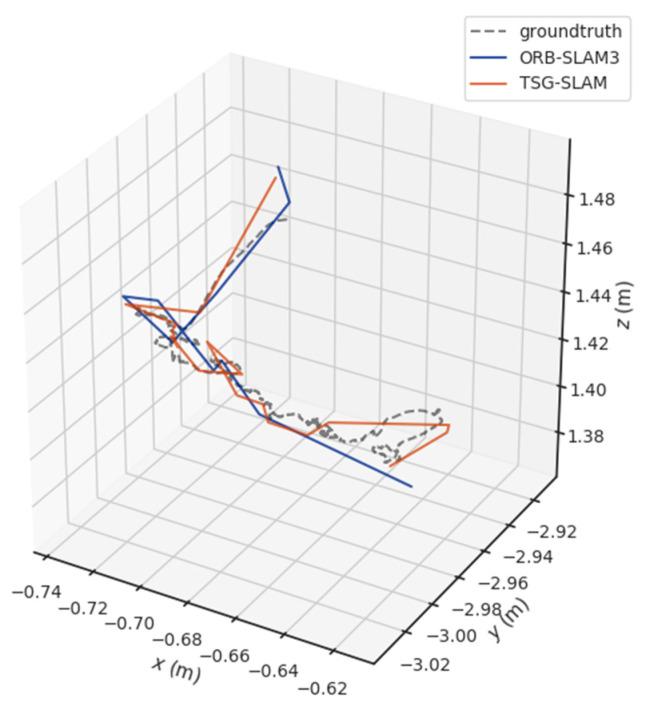
Estimated trajectory vs. true trajectory for fr3/sitting_static.

**Figure 15:**
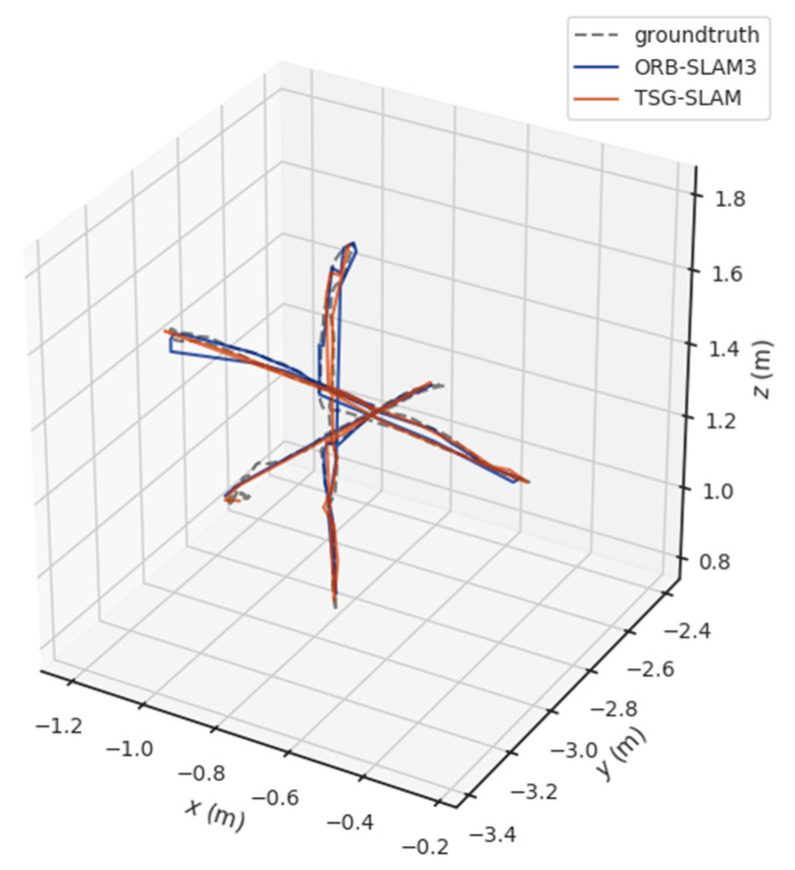
Estimated trajectory vs. true trajectory for fr3/sitting_xyz.

Table 3 compares ATE and RPE in low-dynamic scenarios. In the fr3/sitting_static sequence that focuses on dynamic targets, TSG-SLAM has smaller errors compared to ORB-SLAM3, with RMSE improvement rates of 45.45% and 34.05% for ATE and RPE, respectively. Similarly, in the fr3/sitting_xyz sequence with a larger field of view, the RMSE improvement rates also reach 39.2% and 20.71% for ATE and RPE, respectively. Figure 14 and Figure 15 show a comparison of estimated and true trajectories for the fr3/sitting_static and fr3/sitting_xyz sequences, respectively. ORB-SLAM3 exhibits a certain deviation between the estimated and true trajectories, especially for the fr3/sitting_static sequence, while TSG-SLAM’s estimated trajectory is much closer to the true trajectory. Therefore, in low-dynamic scenarios, TSG-SLAM has a definite advantage in localization, and its localization accuracy is significantly improved.

**Figure 16:**
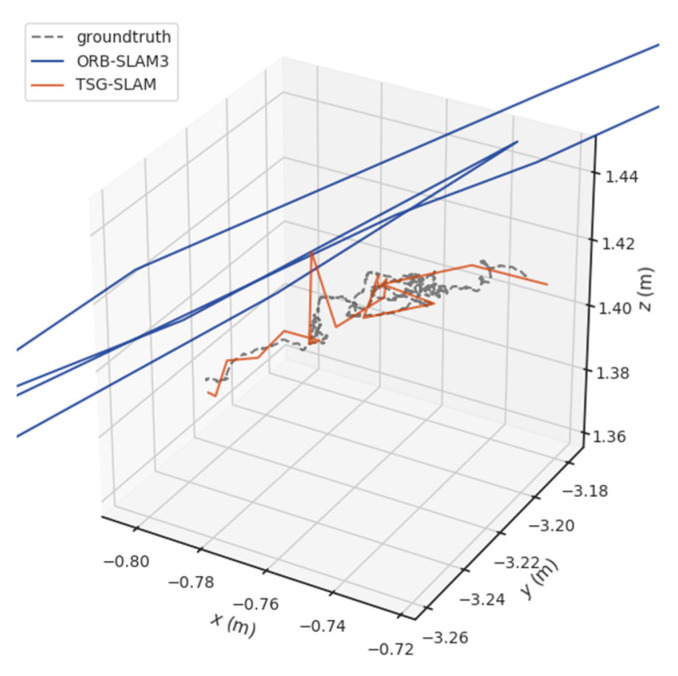
Estimated trajectory vs. true trajectory for fr3/walking_static.

**Figure 17:**
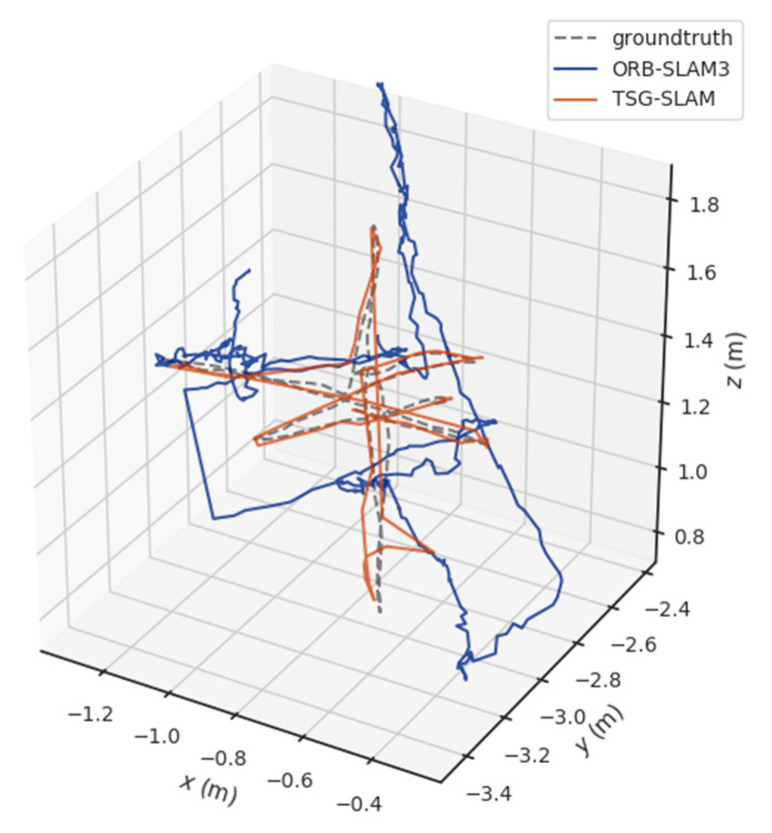
Estimated trajectory vs. true trajectory for fr3/walking_xyz.

Table 4 compares ATE and RPE in high-dynamic scenarios. The errors of ORB-SLAM3 are significant, especially the RMSE of ATE, reaching 0.3832 m and 0.7123 m for the fr3/sitting_static and fr3/sitting_xyz sequences, respectively. In contrast, TSG-SLAM controls the errors well, with both the RMSE and STD improvement rates of ATE exceeding 96%. This indicates that TSG-SLAM has greatly improved global localization accuracy and stability in high dynamic scenarios, and the RMSE and STD improvement rates of RPE also exceed 55%. Figure 16 and Figure 17 show a comparison of estimated and true trajectories for the fr3/sitting_static and fr3/sitting_xyz sequences, respectively. The estimated trajectory of ORB-SLAM3 exhibits significant deviation from the true trajectory, while the estimated trajectory of TSG-SLAM has some deviation from the true trajectory, but they are still relatively close overall. Therefore, in high-dynamic scenarios, ORB-SLAM3 is unable to function effectively, while TSG-SLAM can still function stably and has significantly improved localization accuracy.

To evaluate the superior localization performance of TSG-SLAM in complex environments with dynamic objects, we compared it with several dynamic SLAM systems that have shown good performance in recent years, such as DS-SLAM, DynaSLAM, MISD-SLAM, and RDS-SLAM. Since we used different computers for testing, we could not directly compare the error data obtained. Therefore, we used the relative accuracy improvement rates of these dynamic SLAM systems compared to ORB-SLAM3 as the evaluation standard for a performance comparison, specifically the RMSE and STD improvement rates of ATE.

The comparison results are presented in Table 5. In the low-dynamic scenarios of fr3/sitting_static, TSG-SLAM demonstrated a significant advantage compared to other dynamic SLAM systems. The ATE improvement rate was much higher than that of the other dynamic SLAM systems thanks to the high dynamic segmentation accuracy of the dynamic feature removal method proposed in this paper. The data for DynaSLAM are not provided in the relevant paper; therefore, no comparison could be made. In high-dynamic scenarios, all dynamic SLAM systems showed significant improvements compared to the ORB-SLAM3 system. Although TSG-SLAM had slightly lower improvement rates than some dynamic SLAM systems, it still had certain advantages overall.

In summary, the TSG-SLAM system overcomes the challenges posed by moving targets in complex environments and demonstrates reliable performance in various dynamic environments with high localization accuracy and stability. It also performs comparably to other top-performing dynamic SLAM systems in certain low-dynamic scenarios and even outperforms them in terms of localization accuracy.

**Figure 18:**
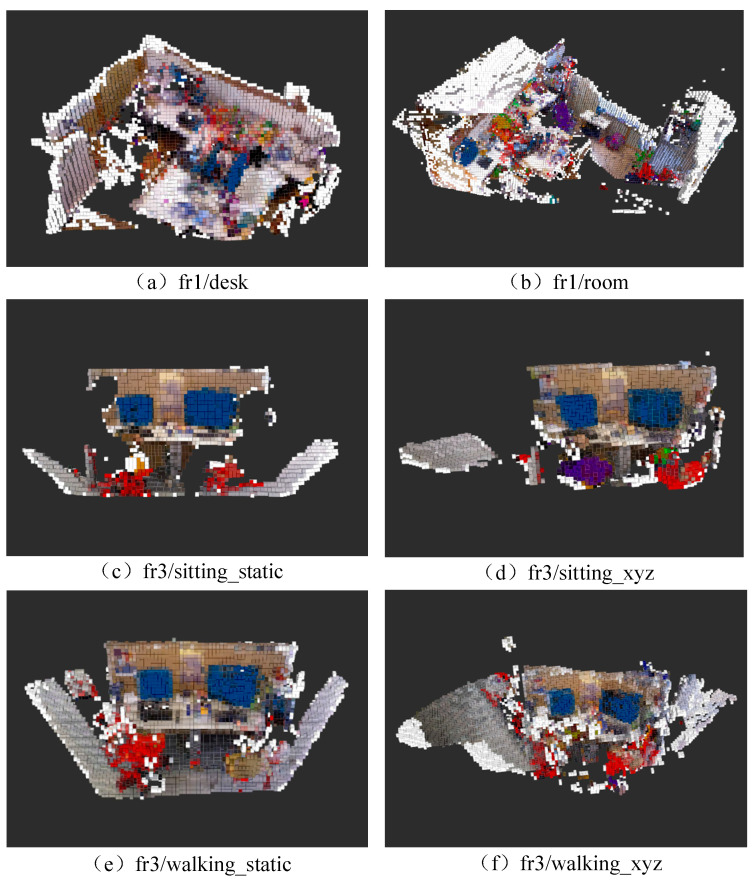
Octree semantic map construction results.

An experimental evaluation was conducted to assess the instance-level semantic mapping performance of TSG-SLAM in static, low-dynamic, and high-dynamic scenarios. Figure 18 depicts the results of scene semantic mapping on six datasets, including fr1/desk, fr1/room, fr3/sitting_static, fr3/sitting_xyz, fr3/walking_static, and fr3/walking_xyz. TSG-SLAM successfully constructs 3D geometric models of the objects in the scenarios and adds semantic tag color information for the objects, which enhances the map’s visualization, such as the blue screen, red chair, and gray table.

Figure 18a,b show the results of semantic map construction for the indoor static scenario with the desktop as the main object and the larger global static scenario, respectively. The scenarios and targets appearing in the sequence were accurately reconstructed, and semantic color information was added to the targets.

Figure 18c,d demonstrate the results of semantic map construction for low-dynamic scenarios. It can be observed that even two people only slightly moving their hands and heads were successfully segmented and most of the features on their bodies were removed, leaving only the static targets for reconstructing the scenario. Additionally, Figure 18c has a smaller reconstruction range than Figure 18d but with better results due to capturing more data from the local screen area caused by a smaller camera movement range. Meanwhile, the camera’s view in Figure 18c is limited, and some scenarios blocked by people were not reconstructed and remain blank, while most of the scenarios blocked by moving people in Figure 18d were reconstructed.

Figure 18e,f depict the results of constructing semantic maps for high-dynamic scenarios. It can be seen that the features of the two moving persons were removed, and the parts of the scenario occluded by people were also reconstructed. The overall scenario reconstruction is relatively complete. The camera motion in Figure 18e is slower and has a smaller range, so the reconstruction scope is smaller, but the reconstruction effect is better.

Therefore, TSG-SLAM, with the help of its dynamic feature segmentation module, is capable of effectively handling the presence of dynamic objects in both static and dynamic scenarios, reconstructing scenarios accurately, acquiring the instance-level semantic information of objects, and building a static 3D semantic map with dynamic interference removed.

### 6.5. Real-World-Scenario Experiments

**Figure 19:**
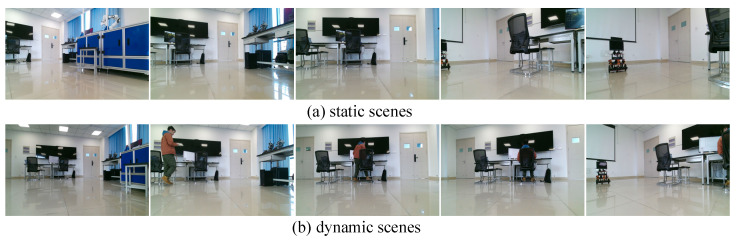
Partial image sequence of real scenario.

In order to verify the effectiveness of the TSG-SLAM system in real-world scenarios, experiments on the simultaneous localization and semantic mapping of mobile robots were conducted in an indoor laboratory. Due to the challenge of replicating identical trajectories for a mobile robot, an experiment was conducted using a mobile robot that was remotely controlled to move around in an indoor laboratory. A dataset was generated with the camera’s viewpoint identical to that of the robot’s movements which was used to evaluate the ORB-SLAM3 and TSG-SLAM systems. The evaluation was carried out on two different experimental scenarios, one static and the other dynamic, to assess the system’s performance in simultaneous localization and semantic mapping. To simulate a dynamic environment, the experimenter moved freely around the scenario, capturing image sequences from various angles, as shown in Figure 19.

In real-world scenarios, it is challenging to obtain an accurate camera motion trajectory. Therefore, the estimated trajectories of TSG-SLAM and ORB-SLAM3 were compared based on the dataset captured by the mobile robot in the experimental scenario. As the ground upon which the mobile robot moves is nearly horizontal, a 2D estimated trajectory plot in the x-y direction was created to facilitate the comparison of the estimated trajectories.

**Figure 20:**
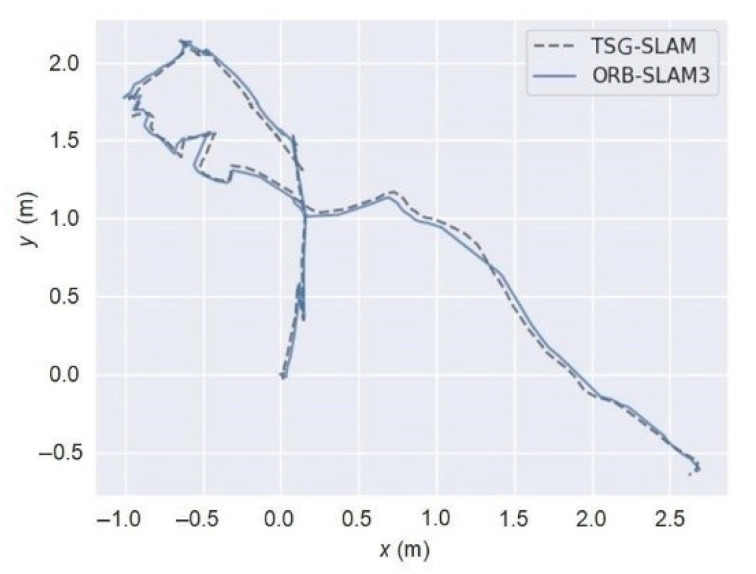
Comparison of estimated trajectory for real static scenarios.

Figure 20 presents a comparison of the trajectory estimation between TSG-SLAM and ORB-SLAM3 in static scenarios. The use of Mecanum wheels with differential steering for the mobile robot can cause jitter in the camera when the steering angle is large, leading to more fluctuations in the estimated trajectory during sharp turns. Nevertheless, the estimated trajectories of TSG-SLAM and ORB-SLAM3 in real static scenarios are nearly identical, which is consistent with the comparison results of the estimated trajectories in public static datasets. As ORB-SLAM3 has good localization accuracy in static scenarios, this result indicates that TSG-SLAM also performs well in real static scenarios.

**Figure 21:**
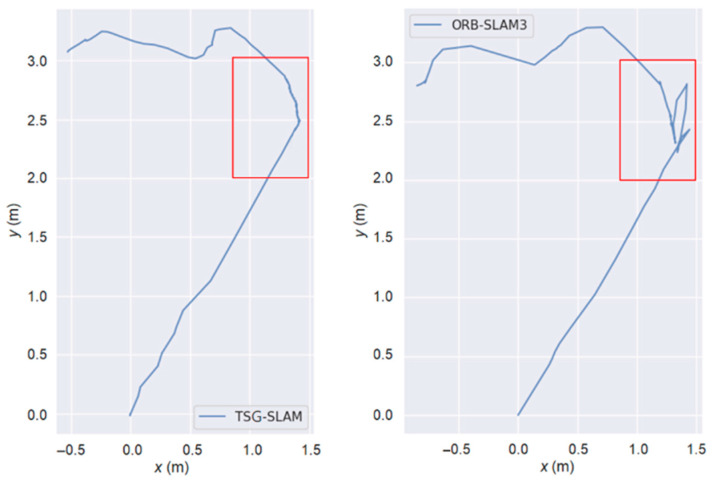
Comparison of estimated trajectory for real dynamic scenarios.

Figure 21 illustrates a comparison of the trajectory estimation between TSG-SLAM and ORB-SLAM3 in dynamic scenarios. In the first half of the trajectory, where no dynamic targets are observed or are still far away from the camera, both methods produce almost identical trajectory estimations. However, in the middle section, where the camera approaches the dynamic target (marked with a red box in Figure 21), ORB-SLAM3 is significantly affected, resulting in substantial fluctuations in the estimated motion trajectory. On the other hand, TSG-SLAM, which processes the dynamic target, is less affected, leading to smaller fluctuations in the estimated trajectory.

**Figure 22:**
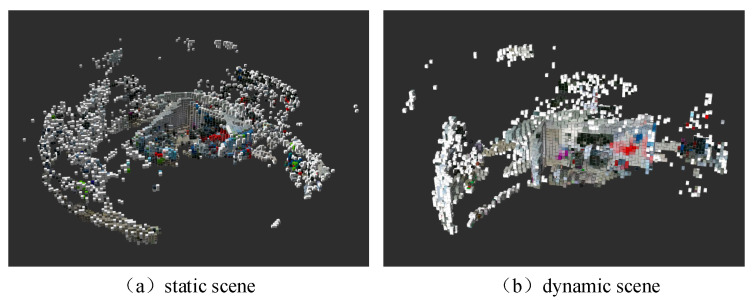
Semantic map of real scenario.

Figure 22 depicts a semantic octree map in a real scenario. Figure 22a,b display the map reconstruction results in static and dynamic scenarios, respectively. Due to the large scenario size and limited data collection, some details are still missing in the semantic map. Nevertheless, the overall effect is impressive, and the 3D geometric models of objects in the actual scenario were established well, with semantic tags and color information added, such as black displays and red tables, which produce a good visualization effect. Figure 22b indicates that TSG-SLAM only reconstructed static targets, while the moving experimenters were not reconstructed. This demonstrates that the dynamic feature module of TSG-SLAM successfully removed the features of dynamic targets, validating the effectiveness of the semantic mapping of TSG-SLAM in real-world dynamic environments.

## 7. Conclusions

This paper introduces TSG-SLAM, a simultaneous localization and semantic mapping method tailored for complex environments. The approach aims to address the impact of dynamic objects on mapping accuracy and the demand for semantic mapping in mobile robots. TSG-SLAM adds two threads to ORB-SLAM3’s three-thread structure: dynamic feature removal and semantic map construction. The dynamic feature removal module tightly integrates the SOLOv2 instance segmentation algorithm with multi-view geometry techniques to detect and eliminate dynamic features, mitigating the influence of dynamic objects on visual SLAM systems. The semantic map construction module fuses target semantic information obtained by the instance segmentation algorithm with the 3D semantic point cloud, creating a 3D octree semantic map containing instance-level semantic information. Experimental results from the use of both public datasets and real-world scenarios demonstrate that TSG-SLAM can counteract the effects of moving objects on localization, exhibit excellent adaptability to dynamic environments, and ensure high localization accuracy and stability. The efficacy of the TSG-SLAM system’s 3D semantic mapping is also validated, providing a theoretical foundation for mobile robots to execute high-level tasks, such as navigation and interaction in complex environments.

Future work is anticipated to focus on three key areas. Firstly, to mitigate the impact on system efficiency, the exploration of lightweight processing methods is proposed to improve segmentation speed and ensure high real-time performance. Secondly, to enhance localization and semantic mapping accuracy, the integration of depth information is suggested, addressing the limitations of the current 2D-based instance segmentation algorithm. Lastly, a more detailed analysis and testing will be conducted on the impact of the quantity and movement patterns of dynamic objects on the system.

## Figures and Tables

**Table 1:** Comparison of the number of different types of feature points.

Dynamic Feature Removal Method	Type of Feature Points		
	Static	Dynamic	Potentially Dynamic
ORB feature extraction	144	54	15
Instance segmentation	144	0	15
Tightly coupled method	144	0	0

**Table 2:** Comparison of ATE and RPE in static scenarios.

Evaluation Index		fr1/Desk			fr1/Room		
		ORB-SLAM3	TSG-SLAM	IR	ORB-SLAM3	TSG-SLAM	IR
ATE (m)	Mean	0.0178	0.0160	10.11%	0.0579	0.0512	11.57%
	Median	0.0144	0.0132	8.33%	0.0468	0.0415	11.32%
	RMSE	0.0212	0.0191	9.91%	0.0660	0.0589	10.76%
	STD	0.0114	0.0105	7.90%	0.0318	0.0297	6.60%
RPE (m)	Mean	0.0144	0.0140	2.78%	0.0146	0.0149	−2.05%
	Median	0.0097	0.0102	−5.15%	0.0113	0.0117	−3.54%
	RMSE	0.0196	0.0192	2.04%	0.0189	0.0192	−1.59%
	STD	0.0134	0.0131	2.24%	0.0119	0.0123	−3.36%

**Table 3:** Comparison of ATE and RPE in low dynamic scenarios.

Evaluation Index		fr3/sitting_static			fr3/sitting_xyz		
		ORB-SLAM3	TSG-SLAM	IR	ORB-SLAM3	TSG-SLAM	IR
ATE (m)	Mean	0.0143	0.0074	48.25%	0.0105	0.0064	39.05%
	Median	0.0133	0.0060	54.89%	0.0088	0.0046	47.73%
	RMSE	0.0154	0.0084	45.45%	0.0125	0.0076	39.2%
	STD	0.0058	0.0039	32.76%	0.0067	0.0049	26.86%
RPE (m)	Mean	0.0174	0.0105	39.66%	0.0161	0.0129	19.88%
	Median	0.0168	0.0087	48.21%	0.0125	0.0096	23.2%
	RMSE	0.0185	0.0122	34.05%	0.0198	0.0157	20.71%
	STD	0.0161	0.0112	30.43%	0.0116	0.009	22.41%

**Table 4:** Comparison of ATE and RPE in high-dynamic scenarios.

Evaluation Index		fr3/walking_static			fr3/walking_xyz		
		ORB-SLAM3	TSG-SLAM	IR	ORB-SLAM3	TSG-SLAM	IR
ATE (m)	Mean	0.3697	0.0054	98.54%	0.5975	0.0228	96.18%
	Median	0.3534	0.0046	98.70%	0.5835	0.0171	97.07%
	RMSE	0.3832	0.0010	99.74%	0.7123	0.0121	98.30%
	STD	0.1009	0.0024	97.62%	0.3877	0.0121	96.87%
RPE (m)	Mean	0.0212	0.0074	65.09%	0.0311	0.0251	19.29%
	Median	0.0093	0.0049	47.31%	0.0207	0.0163	21.26%
	RMSE	0.0367	0.0094	74.39%	0.0850	0.0376	55.76%
	STD	0.0300	0.0059	80.33%	0.0790	0.0280	64.56%

**Table 5:** Comparison of ATE improvement rates of all SLAM system relative to ORB-SLAM3.

Dataset	Index	ATE Improvement Rate				
		DS-SLAM	Dyna SLAM	MISD-SLAM	RDS-SLAM	TSG-SLAM
fr3/walking_static	RMSE	97.76%	98.11%	63.31%	97.78%	99.74%
	STD	97.83%	97.89%	68.92%	97.37%	97.62%
fr3/walking_xyz	RMSE	97.30%	98.21%	95.54%	98.39%	98.30%
	STD	96.69%	98.23%	94.89%	98.52%	96.87%
fr3/sitting_static	RMSE	27.78%	-	11.94%	30%	45.45%
	STD	23.26%	-	24.23%	25.58%	32.76%

## Data Availability

The data that support the findings of this study are available from the corresponding author upon reasonable request. “COCO dataset” at https://cocodataset.org (accessed on 30 January 2023). “TUM dataset” at https://vision.in.tum.de/data/datasets/rgbd-dataset/download (accessed on 30 January 2023).
